# Strategies to improve the magnetic resonance imaging experience for autistic individuals: a cross-sectional study exploring parents and carers’ experiences

**DOI:** 10.1186/s12913-023-10333-w

**Published:** 2023-12-07

**Authors:** Nikolaos Stogiannos, Georgia Pavlopoulou, Chris Papadopoulos, Gemma Walsh, Ben Potts, Sarah Moqbel, Antigoni Gkaravella, Jonathan McNulty, Clare Simcock, Sebastian Gaigg, Dermot Bowler, Keith Marais, Karen Cleaver, Jane Harvey Lloyd, Cláudia Sá dos Reis, Christina Malamateniou

**Affiliations:** 1 Department of Midwifery & Radiography, School of Health and Psychological Sciences, City, University of London, London, UK; 2 Medical Imaging Department, Corfu General Hospital, Corfu, Greece; 3 Department of Psychology and Human Development, University College London, Institute of Education Group for Research in Relationships in NeuroDiversity–GRRAND, London, UK; 4 Anna Freud National Centre for Children and Families, London, UK; 5 Institute for Health Research, University of Bedfordshire, Putteridge Bury Campus, Luton, UK; 6 Southampton General Hospital, University Hospitals Southampton Foundation Trust, Southampton, UK; 7 School of Medicine, Health Sciences Centre, University College Dublin, Dublin, Ireland; 8 Institute of Child Health, Great Ormond Street Hospital for Children NHS Foundation Trust, University College London, London, UK; 9 Community Involvement, University of London, London, UK; 10 Faculty of Education, Health & Human Sciences, University of Greenwich, London, UK; 11 Department of Specialist Science Education, University of Leeds, Leeds, UK; 12 School of Health Sciences (HESAV), University of Applied Sciences Western Switzerland (HES- SO), Lausanne, CH Switzerland; 13 Department of Psychology, School of Health and Psychological Sciences, City, University of London, London, UK

**Keywords:** Autism, Magnetic resonance imaging, Radiography, Healthcare accessibility, Parents, Carers, Inclusivity

## Abstract

**Background:**

Autistic individuals encounter numerous barriers in accessing healthcare, including communication difficulties, sensory sensitivities, and a lack of appropriate adjustments. These issues are particularly acute during MRI scans, which involve confined spaces, loud noises, and the necessity to remain still. There remains no unified approach to preparing autistic individuals for MRI procedures.

**Methods:**

A cross-sectional online survey was conducted with parents and carers of autistic individuals in the UK to explore their experiences, barriers, and recommendations concerning MRI scans. The survey collected demographic information and experiential accounts of previous MRI procedures. Quantitative data were analysed descriptively, while key themes were identified within the qualitative data through inductive thematic analysis.

**Results:**

Sixteen parents/carers participated. The majority reported difficulties with communication, inadequate pre-scan preparation, and insufficient adjustments during MRI scans for their autistic children. Key barriers included an overwhelming sensory environment, radiographers’ limited understanding of autism, and anxiety stemming from uncertainties about the procedure. Recommended improvements encompassed accessible communication, pre-visit familiarisation, noise-reduction and sensory adaptations, staff training on autism, and greater flexibility to meet individual needs.

**Conclusions:**

There is an urgent need to enhance MRI experiences for autistic individuals. This can be achieved through improved staff knowledge, effective communication strategies, thorough pre-scan preparation, and tailored reasonable adjustments. Co-producing clear MRI guidelines with the autism community could standardise sensitive practices. An individualised approach is crucial for reducing anxiety and facilitating participation. Empowering radiographers through autism-specific education and incorporating insights from autistic individuals and their families could transform MRI experiences and outcomes.

## Background

Autistic people often face numerous challenges when accessing healthcare, exacerbated by limited adjustments, unavailability of necessary support, and disparities in professionals’ understanding, knowledge and skills [1, 2]. These difficulties can result in traumatic experiences and ultimately in very poor healthcare outcomes, reducing the quality of life and life expectancy for the autistic population [3, 4]. Autistic people frequently encounter sensory overload in healthcare environments, and issues like clinical procedures may be misunderstood due to communication barriers [5–8], as well as misconceptions and communication problems from and between staff that impact on coordination and service quality [1]. While sensory processing differences are recognised in diagnostic criteria for autism, there remains gaps in understanding and adapting these differences in many professional settings. For example, a study of NHS England-funded in-patient mental health facilities for young people highlighted the significant sensory challenges autistic children and young people face in such settings, which can impede wellbeing and potentially worsen mental health conditions [9]. This underscores the importance of considering sensory-friendly adaptations in healthcare settings to support the needs of autistic people, which is a crucial consideration for improving service quality and patient experience.

Deficiencies in healthcare professionals’ knowledge and skills about autism significantly contribute to the inequalities that autistic people face in healthcare [10–12]. For instance, Unigwe et al. (2017) [13] found that 39% of a sample of 304 UK-based General Practitioners lacked any formal autism training, impacting their ability to provide effective and appropriate care for their autistic patients. Although, it should be recognised that NHS England have taken steps to improve training, such as introducing the Oliver McGowan mandatory training [14], the National Autism Training Programme [15] and A for Adjustment [16]. However, the inaccessibility of healthcare facilities and the absence of suitable adjustments also contribute to the challenges as highlighted by Brice and colleagues [17]. In their study of 537 UK-based autistic adults, the necessary adjustments and accommodations were routinely lacking in both mental and physical healthcare services [17].

The utilisation of Magnetic Resonance Imaging (MRI), an essential medical imaging tool for diagnosing various health conditions [18], presents unique challenges for autistic individuals. The MRI environment, characterised by its confined space and loud acoustic noise [19–21], can induce significant distress, leading to potential reluctance and anxiety towards the procedure [22]. It also requires people to remain still for a prolonged length of time to acquire images with adequate quality, suitable for clinical interpretation. These factors may be particularly challenging for autistic individuals, who may find comfort in specific movements, stimming, or have heightened anxiety and/or sensitivity to the wide-ranging sensory experience of an MRI.

Various approaches to mitigate these difficulties have been developed, including communication adaptations, MRI familiarisation techniques, performing MRI during natural sleep, and the involvement of informal carers and family [11, 23–26]. Yet, the unique nature of the MRI environment may still require further specific adjustments [27–29]. There is also a need for additional research on the feasibility and effectiveness of these methods and for exploring further approaches that enable individualised care adjustments for autistic individuals.

There is currently no unified, evidence-based approach specifically designed to prepare autistic individuals for MRI procedures [11]. It has been suggested that personalised adaptations, which recognise and respect neurodiversity, could be instrumental in improving the healthcare experience and ensuring effective medical image acquisition for this population [30]. The recent COVID-19 pandemic amplified the necessity for such adaptations, having imposed additional complications on hospital visits for autistic individuals, such as limiting the available services and restricting who or how many caregivers can visit [31–33]. In light of such situations, the importance of comprehending the experiences of service users and their families becomes increasingly clear. It is fundamental that these insights are integrated into healthcare delivery and service design to drive meaningful improvements [34]. Some autistic individuals face additional barriers to giving feedback on their experiences. In the case of this, people who provide care for them can be valuable proxies.

This study aims to address this gap by focusing on the perspectives of parents and carers of autistic individuals under 18 years of age. The term ‘autistic’ in this study is inclusive of those that are verbal or non-verbal, and with or without a learning disability. The objectives were to explore the UK autistic children and young peoples’ MRI scan experiences, identify perceived barriers and enablers for a successful and safe MRI examination, and inform future recommendations for practice improvement.

## Methods

### Methodology

Adopting Pavlopoulou’s (2020) [35] ‘experience-sensitive approach’ and aligning with the neurodiversity model, a cross-sectional survey was carried out to gather qualitative data through an online platform. This survey included questions soliciting participants’ demographic data and encouraging open-ended responses. Reporting of the study was based on a mixture of the STROBE guidelines (for cross-sectional studies) [36], COREQ for qualitative aspects of analysis and interpretations [37], and CHERRIES reporting the results of the online surveys [38].

### Participants

The 16 participants were recruited using purposive sampling. The eligibility criteria were (a) parents or informal carers of autistic individuals, (b) who have had a prior MRI experience with their autistic child in the UK, and (c) capacity to consent to the study. While the information and consent form stated that autistic people could participate directly or with the assistance of their parents/carers, it transpired that all of the participants were parents/carers.

### Materials

An online survey, created on Qualtrics (Qualtrics, Provo, Utah, USA) and composing of both closed and open-ended questions was employed. Adaptive questioning was used to manage the complexity and quantity of the questions [38]. The questions, informed by a literature review by Stogiannos et al., (2022) [11], underwent a refining process with feedback from autistic service users, consultants, and medical imaging researchers. The finalised 34-item long survey covered four domains: (i) participant demographics, (ii) MRI specifics (number of scans, duration, anatomy examined, referral reason), (iii) MRI experience and related accommodations, and (iv) recommendations for MRI practice improvements. The survey, utilising neurodiversity-friendly language, was first piloted with service users (n = 4) to ensure clarity, layout, and flow. The feedback from this pilot phase confirmed that the design of the survey was well-received and that no modifications were necessary. This affirmation from autistic service users was key in proceeding with the survey as planned, reinforcing the acceptability and appropriateness of both the content and presentation.

### Data collection

Electronic distribution of the survey lasted from 17th February to 30th April 2021, and it was conducted via various platforms, including the London Autism Group Charity, researchers’ professional networks, and the autistic community on X (formerly Twitter), LinkedIn and Facebook. Reminders and regular re-posts were used to encourage uptake [39].

### Community involvement

From the onset to the conclusion, autistic individuals played an active role as co-producers in the project. They contributed to all aspects, including survey co-production, recruitment, results discussion, and post-study considerations. The project team was also composed of autistic service users as well as radiographers and a nurse who offered their professional perspective on the practicality and logistics of the MRI process.

Adhering to AASPIRE’s guidelines [40], the co-production process allowed for adequate processing time, promoted transparency and power sharing, and aimed at collective dissemination of findings through online interactions.

Social media also played a critical role in gathering wider community input, with announcements and hashtags like *#actuallyautistic* and *#autismfriendlymri* on platforms like X and channels operated by the London Autism Group Charity.

### Ethics

Ethical approvalwas received from the City University of London’s Ethics Committee of the study’s host institution (ETH2021-0950). Electronic informed consent was obtained at the beginning of the survey via a dedicated question, and participants had access to a detailed information sheet and an enquiry email address. Anonymity was upheld throughout, while data collection and data storage followed the research institution’s protocols. Adherence to GDPR principles and institutional protocols was maintained throughout the study.

### Data analysis

#### Survey analysis

The analysis of quantitative data involved calculating the frequency and percentages of survey responses. Descriptive statistical analyses were performed using Microsoft Excel Office 365 (Microsoft Corporation). Participant background demographic and MRI experience data can be seen in Table 1. During the data analysis, it was identified that a small amount of missing data was present. Two participants did not disclose information on learning disabilities, possibly due to uncertainty. Additionally, two participants omitted details regarding past MRI duration and frequency, which may be attributed to their lack of certainty in their recollection.

**Table 1:** Service user demographic characteristics and background specifics from the survey responses

Variable	n (%)*
Responder Variable	
**Category of Respondent**	
Parent Carer	15 (94) 1 (6)
Autistic Individual Variables	
**Sex**	
Female Male	1 (6) 15 (94)
**Age (years)**	
≤ 10 11–17 18–30 ≥ 31	7 (44) 4 (25) 4 (25) 1 (6)
**Learning Disability**	
Yes No	12 (86) 2 (14)
**MRI Duration (minutes)**	
10–20 20–30 > 30	4 (29) 4 (29) 6 (43)
**Number of Previous MRI Examinations**	
1 2+	10 (71) 4 (29)
**Scan Type**	
Head Other	10 (63) 6 (37)

* Response rates may not add up to *n* = 16 due to incomplete data

Qualitative data was analysed using an inductive approach that aligns with what Braun & Clarke (2019) [41] refer to as ‘Code reliability Thematic Analysis’. Following data immersion, initial codes were developed based on participants’ narratives using NVivo Software (QSR International Pty Ltd). These codes facilitated the identification of emergent themes within individual narratives. During this process, the methodological challenge of categorising participant quotes that resonated with multiple themes emerged. To address this, consideration was made based on the primary context within each participant’s narrative, enabling the quotes to be assigned to the most fitting themes. This step was crucial in ensuring that each quote was placed where it would most effectively illustrate the respective theme and contribute to the overall coherence of the findings. Subsequently, a ‘cross-analysis’ was conducted, which entailed systematically comparing these emergent themes across different participants’ narratives to uncover commonalities and divergences. This process helped to synthesise the data into a coherent set of themes that reflected the collective experiences of the participant group. To minimise subjectivity and ensure trustworthiness in our thematic analysis, intra-coder agreement exercises were conducted [42]. These exercises involved the coder revisiting the coding process at multiple intervals over the course of data analysis to ensure consistent application of the codebook. This was achieved by comparing the coder’s application of codes to a random sample of the data set at different points in time, ensuring that the same codes were applied to similar data. The findings from these exercises demonstrated a high level of consistency in the coding application, affirming the reliability of the intra-coder process.

## Results

### Quantitative data

#### Demographic characteristics

A total of 16 completed surveys were analysed, completed by n = 15 parents and n = 1 informal carer of autistic individuals. An Informal carer is someone who gives care but is not a parent or a professional carer. The autistic individuals under their care were mainly males (94% n = 15), under 18 years of age (69% n = 11), and exhibited learning disabilities (86% n = 12) (Table 1).

#### MRI specifics

The results showed that a majority of participants indicated their child or dependent had undergone a single MRI examination (71% n = 10), while 29% reported between 2 and 5 scans (n = 4). As for scan duration, 29% of the dependents underwent scans lasting between 10 and 20 min (n = 4), 29% experienced scans of 20–30 min (n = 4), and 43% had scans exceeding 30 min (n = 6) (Table 1).

### Qualitative data

From the qualitative data, six main themes emerged, encapsulating the experiences and needs of the autistic dependents’ parents and informal carers throughout the MRI process.

### Patient-provider communication

Parents/carers indicated that their child often faced communicative difficulties during an MRI examination due to radiographers not adapting their communication methods to suit the child’s needs. They noted occasions of radiographers failing to use understandable, accessible language that avoids technological jargon, leading to confusion and, occasionally, interruptions due to distress:

The radiographer (….) spoke quickly so son found it very difficult to understand.

Told them to stop talking as it was distressing him more.

When interacting with non-speaking dependents, carers reported a lack of communication tools and strategies from radiographers. Participants also reported that radiographers were not aware that some autistic people may have “*a shorter attention span and difficulties with processing speed*” in which case “*their efforts to communicate with the child ‘may do more harm than good.*’” They attributed this to a lack of appreciation of the diverse communication needs and strengths amongst autistic people. No participants reported any efforts by the radiographers to involve parents in the process of information sharing.

### Pre-procedural preparation

Participants emphasised the importance of advanced preparation for MRI visits, with one stating, “*Preparation can help the child so much*.” They mentioned the potential benefits of providing an information pack before the scheduled examination, offering sufficient time for familiarisation and planning coping strategies:

maybe a pack to be given or sent before the appointment and in plenty of time so they can prepare. It’s important they know how things will go on the day and they know what to expect before the appointment.

should be offered before the day so it is all ready for the day so time can be spent making the person comfortable.

However, most parents reported not receiving preparatory information to assist with the procedure:

*They did not send us any information before the actual MRI scan.*

### Reasonable adjustments

Participants recalled a wide range of both positive and negative experiences related to adjustments made during the MRI scan procedure. High levels of satisfaction were linked with the radiographer’s willingness to help and accommodate their child’s preferences, with one parent illustrating this as:

Asked what music he liked, changed the lighting, removed the tissue paper so he could feel the scan bed, showed him round, offered mirror glasses so he could see me all the time and agreed he could hold his stress balls in his hands.

Another parent described that they were not offered any audio-visual materials as an additional supportive option, and emphasised the need for different options to be readily available:

It wasn’t offered so not sure if this is an option but it should definitely be an option as some people can process and cope better with a movie.

Negative experiences were often connected to inflexibility on the part of the radiographer and lack of understanding of the child’s needs, particularly regarding pandemic-related measures:

The member of staff was very rude, insisting a 7 year old has (sic.) to wear a mask before we even got into the scan room. I tried to explain he was exempt because of his age but also his sensory needs and I had brought his own metal free mask with me but he was still refusing to wear it. I explained the MRI procedure was daunting enough so best we don’t (sic.) push with the mask. He refused to treat my son.

The presence of a parent or relative during the procedure was deemed crucial. When carers were present during the procedure, it helped alleviate anxiety, fostering a sense of security. As experts in their child’s care, parents were able to support with appropriate distraction, positioning, and comforting techniques, as illustrated by the following quotes:

Allowed me in the room, allowed me to explain, allowed me to help sort out the earplugs, pillows, positioning.

I was with my son the whole time.

So, I just held his hand and tried to keep him still during the scan.

However, this was not an experience shared by all parents, with some not allowed to be present:

We were not allowed to be in the MRI area at all.

Carers stressed the significance of having a specialist with knowledge of autism. They personalised the process and shared information using child-friendly examples, which helped alleviate anxiety for both parents and the autistic individual:

A play specialist spoke to him and showed him a video in person before the scan. She also showed him the room and the machine and explained what would happen.

We had a specialist nurse help with the process.

A specialist met with my child in (sic.) arrival and explained MRI via Lego.

### Sensory sensitivities, uncertainty and anxiety

Anxiety related to the hospital visit in general and the MRI examination, in particular, was a frequently reported concern, primarily due to overwhelming sensory experiences. Some children had previous unsuccessful interactions with radiographers, which were traumatic and made subsequent visits extremely challenging.

MRI trauma has made straightforward X-rays difficult and…has provoked…high anxiety moments.

Participants reported various overwhelming experiences during scanning, with the most common being the sounds produced by the scanner. One parent described their child’s experience of the MRI scan noises as unbearable:

He couldn’t tolerate it, he had been totally overwhelmed hearing the scanner.

Other parents also shared similar experiences of sensory overload leading to heightened stress:

He was strapped down, and there were lots of loud noises, which he doesn’t like.

Too noisy, difficult being still, anxiety made breathing difficult.

Additionally, the impact of prolonged waiting times was noted as a significant contributor to stress and exhaustion. One participant described how the unpredictable waiting time served as a source of sensory overload for her child, further exacerbating their anxiety:

It was sensory overload and he had to sit there staring at a big white door for 20 + mins having already waiting (sic.) in the main waiting area for 30 + mins.

### Knowledge and training

Carers voiced concerns about radiographers’ lack of sufficient knowledge about autism. They felt that healthcare providers lacked a comprehensive understanding of autism and how it manifests among autistic individuals. This sentiment is supported by the following quote:

The radiographers came appeared to not really know or understand much about autism, it was obvious that little thought or forward planning had gone into expecting his arrival.

Participants also expressed dissatisfaction with the quality of care provided. They felt that radiographers did not know how to adapt their approach to accommodate the anxieties of an autistic individual, as illustrated in the following quote:

Sadly, clinicians think they know about autism but don’t know enough to carry out fairly anxiety provoking procedure.

Participants also noticed a lack of sensory considerations before and during the procedure and wondered why radiographers had not received autism-specific training to better understand the psychological and sensory profiles of autistic people.

### Parent/carer recommendations

Parents offered many recommendations on how to improve the MRI experience of autistic people. Firstly, they called for enhanced autism-specific training for radiographers, highlighting the lack of expertise and knowledge as a significant challenge:

*Train the staff to deal with neurodiversity with expertise and compassion.*

They also emphasised the need for recognising personal requirements and proposed some practical strategies to help prevent exposure to both visible and hidden stressors:

*Be patient, be understanding and help them to be able to have the scan*.

*Less noise. This was the biggest problem*.

*Please try to see them on time*.

Parents also expressed a desire for radiographers to involve them in the care plan, both before and during the visit, and to acknowledge their expertise and insights on their children. They underscored the necessity for shared decision-making:

*Listen to the parents and carer*.

Some parents thought it would be beneficial for the radiographer to be aware of any special interests of the child and offer access to these before and after the procedure. They also highlighted the importance of offering a tailored, flexible, and individualised, person-centred approach, recognising no one strategy is suitable for all:

*Treat each autistic person differently as each will require different things in order to get through things*.

Finally, they encouraged other parents and caregivers to ask for resources and gather information before their scan, emphasising that preparation is key to a better scanning experience:

*Don’t be afraid to ask questions and prepare before the appointment*.

*Ask for information, tour, speak up - talk through options*.

## Discussion

In this present study, a combination of quantitative and qualitative methods was employed to explore parents’ and informal carers’ experiences of their child or dependent undergoing an MRI scan. The study illuminates a range of findings concerning the experiences of parents and carers of autistic individuals during an MRI examination. It provides further evidence of the necessity for an improvement in the practices of MRI services, underscoring the need for specific adjustments and training needs in the field.

Effective communication emerged as an essential factor in ensuring a successful MRI examination. The study findings corroborate prior research suggesting that the use of accessible language significantly enhances the examination experience for autistic individuals [43, 44]. As corroborated by the current study and others [45], the use of complex, technical language is a barrier that can be avoided. To meet individual communication needs, the incorporation of visual aids could help overcome language and comprehension challenges [46]. With appropriate training, the use of communication aids could be conducted effectively which in turn could boost confidence in working with the radiographer, which should further increase the likelihood of facilitating a successful and appropriate scan [28]. Beyond the examination, the study findings also point to the importance of shared decision-making, echoing previous studies that emphasise the role of parental involvement in healthcare decisions [47].

Pre-procedural preparation emerged as an essential strategy. By gathering knowledge about an individual’s care needs before the appointment [48], radiographers can deliver more personalised care. This knowledge is often missing due to the lack of co-ordination between healthcare services in primary care or lack of a formal autism diagnosis due to delays in appointments. Some autistic people might also choose to mask to avoid stigma associated with autism, of fear this might impact their care. Techniques such as pre-visit phone interviews and environmental familiarisation could further help prepare autistic patients and families. Implementing these strategies could enable radiographers to substantially reduce the anxiety derived from the scanner’s noise, which was identified as a major stressor in the study results. Greater familiarity with the scanner noise, potentially achieved via prior auditory training [49, 50], might also increase the likelihood of conducting successful examinations.

The importance of reasonable adjustments emerged as another prominent theme within our findings. Reasonable adjustments, which are integral to ensuring radiography practice is accessible to autistic individuals [51, 52], encompass a diverse range of strategies. The participants of the present study gave examples, such as a change of lighting, a choice of music, the use of tactile stimuli, support from a specialist nurse or play specialist and the presence of a relative which would enhance patient comfort and improve procedure success. Undertaking such accommodations allows radiographers to more effectively cater to the specific needs of the autistic population, particularly in addressing the challenge of sensory overload. This is a prevalent issue faced by autistic individuals when the sensory environment is not tailored to their unique sensitivities [6]. By adapting the environment and procedures to meet these needs, practitioners can improve the overall experience and outcomes for autistic individuals. It is important to note that for continuity of care, these adjustments should be offered and catered for not only in radiology but holistically at the hospital level, to aid in improving accessibility and reducing the distress of autistic people.

Another key theme in the findings was the need for an improvement in staff knowledge and training on autism. Parents expressed discontent with radiographers’ understanding of autism, reflecting a significant obstacle to a satisfactory MRI experience. These findings align with recent research advocating for autism education packages to be incorporated into healthcare curricula [53]. A more nuanced understanding of autism’s heterogeneity would foster more personalised care and greater flexibility when addressing sensory and communication related needs. Radiographers, like other healthcare professionals, are a workforce that possess considerable empathy and a diverse skillset. However, empathy in isolation is not adequate; specialised training is essential, especially for effectively interacting with autistic patients and appropriately adjusting to their specific needs and preferences. There are now different initiatives to offer training on autism for clinical practitioners, notably the Oliver McGowan mandatory training [14]. However, it must be noted that training has to integrate well within the busy clinical routine of healthcare professionals, particularly considering the workforce is just emerging from the difficult experience of the COVID-19 pandemic. To ensure maximum impact at minimal time commitment, training should offer experiential learning that is delivered by autistic and neurodivergent people themselves.

It is also crucial to develop clear and specific guidelines for MRI procedures for autistic individuals. The creation of these guidelines should not be an isolated process but rather be co-produced, bringing together the expertise and insights of autistic individuals, their family carers, and allies, as well as radiographers and other relevant health care professionals. This collaborative approach can ensure that the guidelines are realistic, applicable, and respectful of the lived experiences of those they are intended to support [11]. These guidelines would not only standardise practices, but also promote radiographer confidence, ensuring a consistent, sensitive, and person-centred approach to care. Furthermore, it is imperative that these guidelines align with the initiatives of other professional bodies that have implemented similar measures [54]. In the interest of inclusivity and impact, it is necessary to make these guidelines accessible to all stakeholders. This accessibility should be ensured by making them available in a range of different formats, such as easy-read versions, visual guides, and audio descriptions. By doing so, we can maximise their reach, ensuring that they are easily comprehended and implemented, ultimately making MRI procedures as inclusive, accessible, and successful as possible for the autistic and wider autism community (i.e. parents, carers, family members and allies).

### Limitations

This study possesses several limitations. First, purposive sampling does not allow generalisation of the findings outside this sub-population. The online survey format potentially limited the depth of qualitative insights, which could have been enriched by more in-depth methods such as focus groups or one-to-one qualitative interviews. The sample size, while adequate for an exploratory study, might not have captured the full heterogeneity of the target population and their experiences. The study also took place during one of the national lockdowns imposed by the COVID-19 pandemic, so this impacted data collection methods and recruitment. The pandemic-specific restrictions and regulations applied to all healthcare settings might have also further contributed to a negative experience reported by autistic service users.

Bias may have been introduced due to self-motivated participation and the study’s dependency on internet access, leading to an underrepresentation of certain groups. A lack of detailed descriptive data further restrained the study’s breadth and replication potential.

Additionally, the reliance on parent and carer perspectives as proxy for their autistic family members may not fully represent the experiences of the autistic individuals themselves. Thus, to achieve a more comprehensive understanding, future studies should aim to include autistic people, with a focus on those who face additional barriers to participating in research. This can be done using methods, such as use of photography [55], arts or creative activities [56] or other alternative communication methods [57]. Furthermore, future research would benefit from adopting a prospective observational or experimental approach to evaluate the impact of various adjustments, for example – changing the sensory environment or using visual aids, in standard clinical settings.

## Conclusion

Navigating the healthcare system can present multifaceted challenges for autistic individuals and their families, often compounded by inadequate communication, environmental adaptations and procedure adjustments. This UK-based survey brings to light significant considerations to enhance the experiences of autistic individuals when accessing MRI services.

Key to patient-centred care in radiography is an individualised approach that includes thorough preparation before procedures, which are sensitively tailored to individual needs. In addition, modifications in the clinical environment, such as minimising waiting periods and sensory stimuli, can effectively alleviate anxiety and foster better participation.

Equally important is the empowerment of radiographers through targeted autism training. This, combined with effective collaboration with the autistic and wider autism community, could enable the provision of flexible, patient-centred accommodations. This will ultimately lead to a comprehensive framework and nationwide guidance, to further improve the experiences for autistic individuals and their families.

## Data Availability

The anonymised dataset generated and analysed during the current study is available from the corresponding author on reasonable request.

## Abbreviations

MRI: Magnetic Resonance Imaging
GDPR: General Data Protection Regulation
COREQ: Consolidated criteria for reporting qualitative research
CHERRIES: Checklist for Reporting Results of Internet E-Surveys
STROBE: Strengthening the Reporting of Observational Studies in Epidemiology
AASPIRE: Autistica’s Adult Autism Spectrum Partnership in Research and Education
UK: United Kingdom
